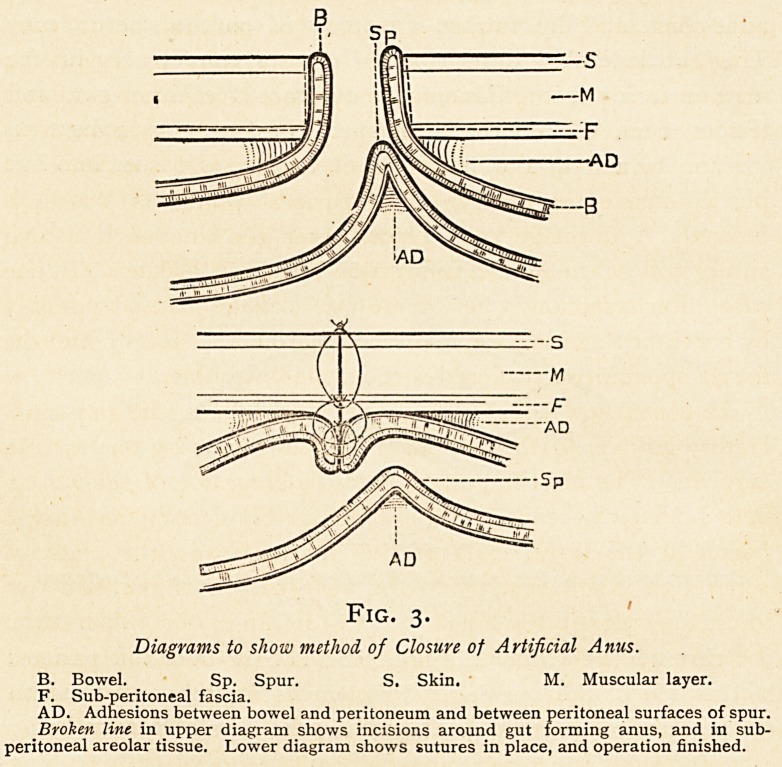# Extra-Peritoneal Closure of Artificial Anus and Fæcal Fistula

**Published:** 1895-03

**Authors:** J. Greig Smith

**Affiliations:** Professor of Surgery, University College, Bristol; Surgeon to the Bristol Royal Infirmary


THE BRISTOL

Medico-Chirurgical Journal.

MARCH, 1895.

### EXTRA-PERITONEAL CLOSURE OF ARTIFICIAL ANUS AND FÆCAL FISTULA.

BY

J. GREIG SMITH, M.B., F.R.S.E.,

*Professor of Surgery, University College, Bristol; Surgeon to the Bristol Royal Infirmary.*

---

DURING the past few years I have had to deal with a number of cases of fæcal fistula left after intestinal drainage in cases of obstruction; and also with some cases of artificial anus left after intestinal resection for malignant disease and for gangrene associated with obstruction. The method I employ is so safe, and has been so uniformly successful, that I think a short description of it may be acceptable.

The aim of the operation is to perform enterorraphy without opening the general peritoneal cavity; and this is managed by detaching from the parietes all round the fistula or anus sufficient peritoneum to permit delivery of the gut through a parietal incision without separating it from its peritoneal adhesions.

It is unnecessary to emphasise the importance of being able to deal with an intestinal fistula without opening the general cavity. This is the main feature underlying the procedure I advocate; and no arguments in its support need be adduced. But in respect of another feature, the approximation of surfaces not covered with peritoneum but simply rawed or covered with cicatrix, for the purpose of closing an opening in intestines, some argument may be necessary.

Too much has been made of the agglutination of peritoneal surfaces in abdominal surgery. It is true that irritated peritoneal surfaces very soon become glued together by lymph. But such union is neither permanent nor strong. It serves a useful purpose by preventing leakage till true union by vascularised granulations takes place. It is by no means certain that two apposed surfaces of intact peritoneum unite as quickly as two surfaces denuded of peritoneum. The process of vascularisation, which is essential to true union, is not likely to be so rapid where a double layer of intact endothelium has to be pierced as where there is no such obstacle. Whether this be true or not, it is certain that in practice the fallacy of peritoneum to peritoneum has been proved again and again, and need not further be insisted upon. Here I do not seek to push this thesis to its full outcome: all I desire to insist upon is, that an opening in gut can be closed as satisfactorily and as speedily by the apposition of rough cicatricial tissue on its surface as by the apposition of intact peritoneum. The operation I suggest brings raw surface to raw, and does not involve peritoneum. There is a sufficiency of proof that it is successful and safe, and there is no need to carry the argument further.

Between the parietal peritoneum and any discharging intestinal opening is a circle of adhesions binding the bowel to the parietes. These adhesions are left intact. The bowel is delivered through an incision carried above and below the parietal opening along with parietal peritoneum, which is separated from parietes to any extent desired. The chief element in the operation is this separation of parietal peritoneum, with its fat, all round the fistula. It is remarkable how much freedom for manipulation a peritoneal stripping of an inch all round will give. A stripping over a circle of two inches radius will permit the gut to be delivered completely through the wound. The detachment is begun at a distance from the fistula, and carried down to it; it may be done almost entirely with the fingers. Further details may now be given.

### Fæcal Fistula.

Here the bowel does not protrude through the parietal opening, and there is no spur, or only a slight one. A simple fistula lined with granulations leads from skin to bowel.

The granulations are first scraped from the sinus by means of a small sharp Volkmann's spoon, and the parts around are purified. If there is any discharge from the intestine, a small sponge with string attached is pushed through the fistula so as to block it.

Fig. 1.

*Diagrams to show method of Closing Fæcal Fistula.*

Fi. Fistula in abdominal wall communicating with bowel.
G. Granulations lining fæcal fistula.
S. Skin. M. Muscular layer. F. Sub-peritoneal fascia.
AD. Adhesions between bowel and peritoneum surrounding fistula. B. Bowel.
*Broken line* in upper diagram shows incisions around fistula and in sub-peritoneal areolar tissue. Lower diagram shows operation finished and sutures placed.

Two incisions are now made in the parietes, with the fistula as centre, down to the sub-peritoneal areolar tissue. Their direction is to be guided by that of the principal muscular fibres in the parietes, so as to avoid their division and thus minimise weakening of the parietes. A fistula in the middle line would have vertical incisions above and below it; in the loin it would be vertical or oblique, as we desire to preserve the fibres of the internal oblique muscle, or external oblique muscle and aponeurosis. The incision comes up to, but does not pass through, the fistula; it is carried round the fistula; the fistula with the cicatricial tissue surrounding it is bodily removed. The parietal incision goes down to the sub-peritoneal areolar and fatty tissue, but does not go through it. Then with finger and scissors the parietal peritoneum with its fat is detached from the muscle all round the fistula for a distance of from one to two inches. When the separation is complete, the fistulous tract is removed down to the gut. The bowel remains attached to the parietal peritoneum by adhesions around the fistulous opening. By means of forceps placed close to the opening, the bowel, with its attached peritoneum, may now be lifted out through the incision in the parietes. If there is any difficulty in doing this, a little more detachment of peritoneum will make it easy. The opening in the gut is now closed by infolding of the rawed areolar surfaces around the fistula and suturing by Lembert's method, as if smooth peritoneal surfaces only were involved. The line of closure may be vertical or transverse, as seems best. Two layers of closely-placed sutures, one continuous (Dupuytren), suffice for closure. The outer row will engage some of the sub-peritoneal areolar tissue, and should have a considerable grip of material. The sutured gut and peritoneum is pushed inside, and the parietal wound closed over it by silkworm gut sutures in the ordinary way. A small drainage tube laid over the line of gut suture adds to the security by preventing burrowing of fluids in case of leakage.

### ARTIFICIAL ANUS.

Here the intestine itself forms the surrounding of the fistula; the mucous membrane of the bowel and the skin are practically continuous. There may be ectropion of intestinal mucous membrane or of the whole bowel. There will always be a spur more or less perfect, and, according to its perfection, requiring previous treatment. If there has been loss of bowel from resection or gangrene the spur will be dense and unyielding, but it need not be so large as when it is made simply by kinking.

In every case where the spur is well developed or where the intestine below the artificial anus is contracted it will be wise to devote a few days before operation to the amelioration of both conditions. For these purposes I have found Mitchell Banks's ingenious method, by means of a piece of rubber tubing, quite efficient. The tubing, if introduced on the stretch, may be of considerable dimensions. It rests comfortably in the large entering gut; dilates the contracted efferent gut, and presses back the encroaching spur (*Fig.* 2). It is kept in position by a loop of aluminium wire, which is passed into but

FIG. 2.

*Diagram to show Banks's method of Reducing Spur and Dilating Contracted Bowel in Artificial Anus.*

not through the wall of the tube, and is bent over the parietes by the side of the opening. This wire, if strong and fixed to the parietes by strapping, will prevent the tube both from being extruded and from being carried down the bowel. The tubing sets up some intestinal catarrh with secretion of mucus, and it may cause some pain. In most cases it can be borne with or without the assistance of opium. In a few days the spur will be reduced, and the lower gut will be dilated. A couple of days' rest may be given to the irritated intestine before operation.

The operation is begun as in that for fistula, by making incisions along the direction of the chief muscular fibres on each side of the opening down to the sub-peritoneal tissue. The length of the incisions will vary according to the thickness of the parietes, but will not be shorter than two inches on each side of the anus. The knife is carried round the gut adherent to the parietes, liberating it thoroughly. The peritoneum, with its areolar tissue, is separated from the overlying muscle all round over a circle of two inches radius or more. The bowel,

FIG. 3.

*Diagrams to show method of Closure of Artificial Anus.*

B. Bowel. Sp. Spur. S. Skin. M. Muscular layer.
F. Sub-peritoneal fascia.
AD. Adhesions between bowel and peritoneum and between peritoneal surfaces of spur.
*Broken line* in upper diagram shows incisions around gut forming anus, and in sub-peritoneal areolar tissue. Lower diagram shows sutures in place, and operation finished.

with its adherent parietal peritoneum, is then delivered through the peritoneum. All superfluous pieces of tissue are removed, and the gut is ready for suture.

Usually union is best made transversely. If there has been resection of gut, transverse suturing is essential. If there has been only incision of bowel, as in colostomy or enterostomy, suture may be longitudinal; but even here it is perhaps best done transversely. I have succeeded equally well by each method.

Sutures are carefully placed by the Lembert method from behind forwards. Particular care is given to the deep suturing. A good hold of the tissues is taken, and each stitch must bring about accurate apposition. A single or double row of sutures is placed over the deep row, and here also perfect closure, without undue compression, must be secured. Tension may be avoided by complete liberation of the bowel from surrounding adhesions, and by further stripping of parietal peritoneum. The gut is closed exactly as in enterorraphy by Lembert's method inside the peritoneum; only there being more available tissue for union a more extensive grip is taken by each suture.

The wound in the bowel is cleansed; and the whole is pushed inside the cavity. The parietal wound is closed as before. A drainage tube placed over the line of intestinal suture will guard against infiltration if there is leakage. In one case (after resection) where there was leakage (caused possibly by constant vomiting for many hours from the anæsthetic) the fistula spontaneously closed without much trouble.

In conclusion, I may add that the operation, safe and satisfactory as it is to the patient, is not a very easy one for the surgeon. The most important detail is liberation of the gut by detachment of the parietal peritoneum. If detachment is well begun at the distal ends of the incisions, and the plane of separation is followed up to the very margin of the fistula or anus, the operation is much simplified. In my first operations I began the detachment from the edges of the fistula; this is not so easy, and may lead to opening of the cavity. Free detachment of parietal peritoneum, with accurate suturing of the bowel, are the most important elements of success.

---

### MODERN SURGICAL TECHNIQUE.

Dr. H. O. Marcy (*Medical Record*, March 2, 1895), says that almost unconsciously we are led to think of modern surgical technique as an *art* rather than a *science*. Let us so accept it. Based upon scientific principles, the work should be artistic; a blundering surgeon may be aseptic, but this should never be an excuse for clumsiness. The true surgeon is skilful, which term means so much, full of that skill which makes his work really correctly automatic. Anæsthesia and asepsis have brought into surgery a small army of men who lack the training which, half a century ago, was considered absolutely essential to success. The greatest danger to-day lies in the willing, enthusiastic recruits with which our ranks are overcrowded.

## Figures and Tables

**Fig. 1. f1:**
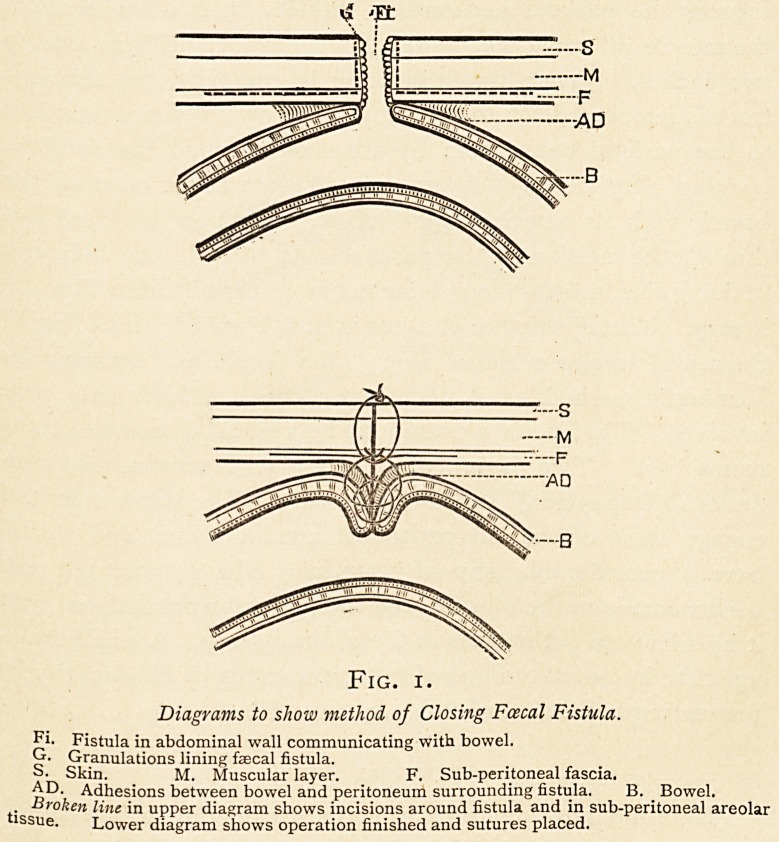


**Fig. 2. f2:**
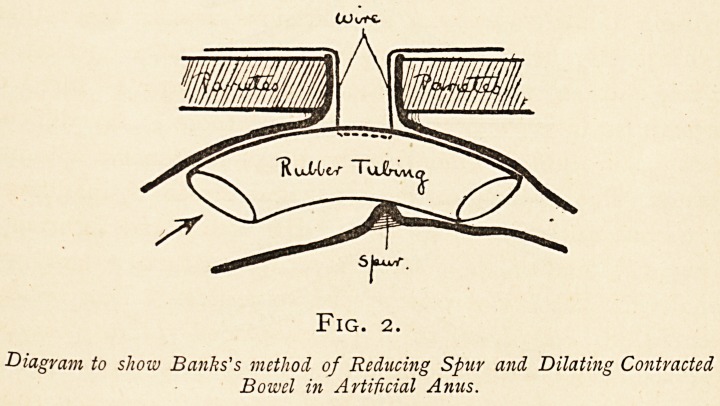


**Fig. 3. f3:**